# The role of diffusion tensor imaging as an objective tool for the assessment of motor function recovery after paraplegia in a naturally-occurring large animal model of spinal cord injury

**DOI:** 10.1186/s12967-018-1630-4

**Published:** 2018-09-17

**Authors:** Adriano Wang-Leandro, Marc K. Hobert, Sabine Kramer, Karl Rohn, Veronika M. Stein, Andrea Tipold

**Affiliations:** 1 Department of Small Animal Medicine and Surgery, University of Veterinary Medicine Hannover, Hannover, Lower Saxony Germany; 2 Centre of Systems Neuroscience, Hannover, Lower Saxony Germany; 3 Institute of Biometry, Epidemiology, and Information Processing, University of Veterinary Medicine Hannover, Hannover, Lower Saxony Germany; 4 Present Address: Department of Diagnostics and Clinical Services, Clinic for Diagnostic Imaging, Vetsuisse Faculty, University of Zürich, Zurich, Switzerland; 5 Present Address: Division of Clinical Neurology, Department of Clinical Veterinary Sciences, Vetsuisse Faculty, University of Bern, Bern, Switzerland

**Keywords:** MRI, Intervertebral disc herniation, IVDH, Hemilaminectomy, Canine, SCI, DTI, Translational medicine, Follow-up studies

## Abstract

**Background:**

Traumatic spinal cord injury (SCI) results in sensory and motor function impairment and may cause a substantial social and economic burden. For the implementation of novel treatment strategies, parallel development of objective tools evaluating spinal cord (SC) integrity during motor function recovery (MFR) is needed. Diffusion tensor imaging (DTI) enables in vivo microstructural assessment of SCI.

**Methods:**

In the current study, temporal evolvement of DTI metrics during MFR were examined; therefore, values of fractional anisotropy (FA) and apparent diffusion coefficient (ADC) were measured in a population of 17 paraplegic dogs with naturally-occurring acute SCI showing MFR within 4 weeks after surgical decompression and compared to 6 control dogs. MRI scans were performed preoperatively and 12 weeks after MFR was observed. DTI metrics were obtained at the lesion epicentre and one SC segment cranially and caudally. Variance analyses were performed to compare values between evaluated localizations in affected dogs and controls and between time points. Correlations between DTI metrics and clinical scores at follow-up examinations were assessed.

**Results:**

Before surgery, FA values at epicentres were higher than caudally (p = 0.0014) and control values (p = 0.0097); ADC values were lower in the epicentre compared to control values (p = 0.0035) and perilesional (p = 0.0448 cranially and p = 0.0433 caudally). In follow-up examinations, no significant differences could be found between DTI values from dogs showing MFR and control dogs. Lower ADC values at epicentres correlated with neurological deficits at follow-up examinations (r = − 0.705; p = 0.0023).

**Conclusions:**

Findings suggest that a tendency to the return of DTI values to the physiological situation after surgical decompression accompanies MFR after SCI in paraplegic dogs. DTI may represent a useful and objective clinical tool for follow-up studies examining in vivo SC recovery in treatment studies.

**Electronic supplementary material:**

The online version of this article (10.1186/s12967-018-1630-4) contains supplementary material, which is available to authorized users.

## Background

Spinal cord injury (SCI), a devastating disease affecting the central nervous system, has a worldwide estimated incidence range in humans from 3.6 to 195.4 cases per million [1]. It involves individual damage of motor and visceral functions and consequently leads to detriments in quality of life and represents a high economic burden [2].

Curative therapies for SCI are currently subject of research and development of techniques that may enable an objective assessment of recovery phases are needed [3–6]. Traditionally, the use of rodent models has been established as a highly-standardized research tool for diagnostic, prognostic, and therapeutic approaches in SCI [3, 7, 8]. However, induced lesions in the rodent spinal cord still evidence large discrepancies in relation to human traumatic SCI concerning pathophysiology, anatomy and histopathology [9, 10]. Therefore, research in large animal models that can bridge the gap between rodents and humans is needed [11]. The dog is increasingly recognized as a large animal translational model for various pathologies of the central nervous system including multiple sclerosis, epilepsy and traumatic SCI [9, 11–17].

Spinal cord injury caused by acute intervertebral disc herniation (IVDH) is one of the most common neurological conditions in dogs [18]. IVDH may occur when biomechanical forces are applied to the nucleus pulposus leading to rupture of the dorsal aspect of the annulus fibrosus and sudden extrusion of degenerated disc material into the vertebral canal [19, 20]. This spontaneous, naturally-occurring, ventro-dorsal herniation induces a mixture of contusive and compressive forces acutely exerted to the spinal cord, strongly resembling human traumatic SCI [9, 11, 21]. Depending on several factors such as the localization of the herniation, degree of compression and amount of material extruded, clinical signs may involve a wide spectrum of neurological deficits varying from mild paravertebral hyperaesthesia to paraplegia without response to nociceptive stimulus [22].

Magnetic resonance imaging (MRI) of the spinal cord remains the gold standard for the diagnosis of canine IVDH [23–25]; however, versatility of this technique allows to transcend beyond diagnostic purposes and provide valuable and objective information concerning integrity of spinal cord parenchyma [26]. Diffusion tensor imaging (DTI) is a modality of MRI that enables in vivo non-invasive tissue characterization by means of water molecule diffusion [27]. Microarchitecture of the nervous system, particularly the white matter, permits homogeneous and direction-dependent water molecule displacement with greater freedom of movement parallel to axonal bundles [27]. This directional dependency, also defined as anisotropy, enables DTI to infer and quantify diffusion behaviour [28]. Fractional anisotropy (FA) and apparent diffusion coefficient (ADC) are commonly reported indexes used for spinal cord DTI [29]. Measurements of FA depict the degree of directionality present within a specific tissue, and are determined by inherent tissue characteristics, for instance myelin, cellular membranes and microtubules [30, 31]. It ranges from 0 to 1, with values close to 0 meaning an unrestricted random diffusion, whereas measurements close to 1 are interpreted as highly restricted or anisotropic diffusion [32]. Furthermore, ADC represents the average magnitude of molecule displacement at any diffusion direction determined [28, 33].

As patient management may represent a restraining factor limiting the time for MRI scans in acute traumatic SCI, the interest for using DTI in the spinal cord of dogs as a large animal model for traumatic SCI has been increasingly growing [34–38]. We recently characterized acute and chronic stages of severe SCI and evaluated the prognostic value of DTI for predicting early MFR [34, 39]. However, description of DTI metrics during MFR may represent a useful tool for objective in vivo evaluation of the spinal cord parenchyma during clinical trials.

The aim of this study is to describe the temporal evolvement of DTI metrics in paraplegic dogs with acute SCI showing MFR after surgical decompression of the spinal cord. We hypothesize that diffusion alterations present during acute, naturally-occurring SCI will not be detectable in dogs showing MFR after decompressive surgery and that DTI metrics at the lesion epicentre measured 12 weeks after MFR will correlate with the clinical status.

## Methods

### SCI dogs

**Fig. 1:**
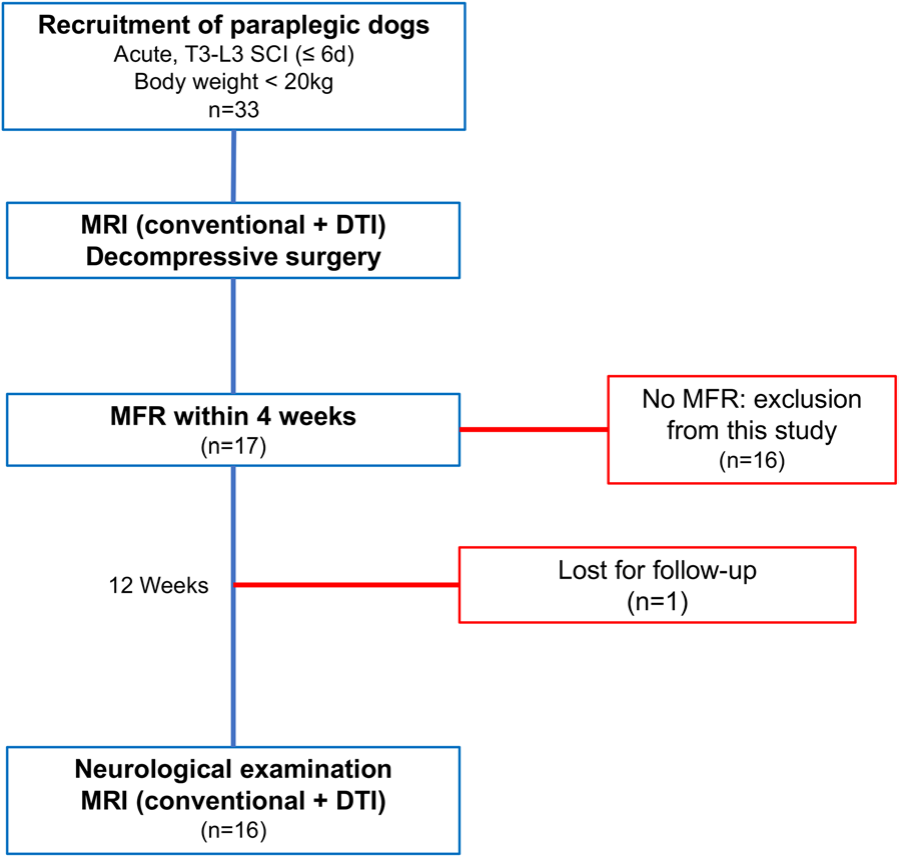
Flow chart illustrating the study design. *DTI* Diffusion tensor imaging, *MFR* motor function recovery, *MRI* magnetic resonance imaging, *SC* spinal cord, *SCI* spinal cord injury

Private owned dogs admitted to the Department of Small Animal Medicine and Surgery, University of Veterinary Medicine Hannover were prospectively recruited in a period between June 2013 and April 2015 with the following inclusion criteria: acute (≤ 6 days) onset of paraplegia consistent with T3-L3 SCI after IVDH with either presence or absence of deep pain perception (DPP), a body weight less than 20 kg and recovery of voluntary motor function within 4 weeks after decompressive surgery (Fig. 1). DPP was tested producing a noxious stimulus, clamping the digits of the hind limbs with forceps. A positive reaction to this test was considered, when an obvious and reproducible behavioural response that could be interpreted as pain was elicited, i.e. whining, turning the head towards the origin of stimulus or attempting to bite [40]. Voluntary motor function recovery was defined as presence of pelvic limb movement evaluated with and without support and intact DPP. Dogs with diagnosis of IVDH or spinal cord compression caudal to the intervertebral space L3/4, showing clinical signs compatible with a lower motor neuron lesion and/or absence of MFR within 4 weeks postoperatively were excluded from the study.

Identifying the exact starting time point of SCI in naturally-occurring IVDH is challenging in dogs. In the current study, the onset of non-ambulatory status as first noticed by the dog’s owner was used to temporally classify and determine the acute stage of SCI [40]. Each SCI dog underwent a general physical and neurological examination, as well as diagnostic imaging consisting of radiographs of thoracic and lumbar vertebral column and MRI of the thoracolumbar spinal cord as described below. Additionally, a complete blood cell count, serum biochemistry analysis, urinalysis and examination of cerebrospinal fluid were performed to exclude several differential diagnoses. Subsequently, the spinal cord was surgically decompressed by hemilaminectomy [41, 42]. Diagnosis of IVDH was confirmed by MRI and presence of herniated intervertebral disc material during surgery. A follow-up neurological exam and MRI scan was performed 12 weeks after MFR was observed.

As controls, six dogs, 5 males and 1 female, with either orthopaedic disease or neurological signs localized outside the T3-L3 segment of the SC were included and already used in another study [35, 39]. Their mean age was 6.4 years (median 6.4 years; range 1.7–12.1 years) and their mean body weight 15.6 kg (median = 11.8 kg; range 6–30 kg). This study was performed after the approval of the German Animal Welfare authorities [Lower Saxony State Office for Consumer Protection and Food Safety (LAVES); Number: 33.9-42502-04-11/0661] and the written owners´ consent for each examination.

### Magnetic resonance imaging

A 3 Tesla MRI scanner (Phillips Achieva, Phillips Medical Systems, Eindhoven, The Netherlands) together with a SENSE (sensitivity encoding)—spine coil with 15 channels was used to perform the examinations.

Each examination was performed under general anaesthesia and artificial ventilation. For premedication either acepromacine (0.05 mg/kg BW IM) or diazepam (0.5 mg/kg BW IV) together with levomethadone (0.2–0.6 mg/kg BW IV) was used. Anaesthesia was induced with propofol (2 mg/kg BW IV) and maintained with isoflurane in air and oxygen. For image acquisition, dogs were placed in dorsal recumbency and at least sagittal and transversal planes of Turbo-Spin-Echo T2-weighted sequences, transversal gradient-echo T2*-weighted for assessment of presence of intramedullary haemorrhages and Echo-Planar-Imaging DWI SE sequences of the thoracolumbar SC were performed.

For the acquisition of T2-weighted (T2W) sagittal images the following protocol parameters were used: TR of 3100 ms with a TE of 120 ms, slice thickness of 1.8 mm, and a slice interval of 0.2 mm. The FOV varied from 301.2 to 392 mm. For transversal planes of the same sequence TR varied from 4630.4 to 8418.8 ms with a TE of 120 ms, slice thickness of 2 mm, a 0.2 mm slice interval and a FOV of 190 mm. Transverse T2* sequences were acquired with a TE of 6.9 and a TR of 520.5 to 662.2 ms; slice thickness of 2 mm, slice interval of 2.2 mm and a FOV of 150 mm.

The DTI protocol consisted of a TR range of 2758.1–11668.8 ms, which varied according to dogs’ size, length of scanned area, and consequently number of slices. TE was 70 ms, a slice thickness of 2.00 mm with no slice interval and a FOV of 214 mm were implemented. Furthermore, 32 diffusion directions were applied, low b value = 0 s/mm^2^, maximal b value = 800 s/mm^2^, and a voxel size of 1.65 × 1.65 × 2.0 mm [43]. To overcome interference with epidural fat, sequences were acquired using spectral presaturation with inversion recovery (SPIR) for fat suppression. Dynamic stabilization was automatically implemented to enhance image consistency and to ameliorate signal drift [44]. A diffusion registration package was applied during acquisition in order to reduce geometrical distortions caused by eddy current induced artefacts [35, 45, 46].

### Methodology

Spinal cord injury dogs were classified according to a standardized clinical 5 grade scale [47], where (0) represents a dog without any neurological deficit indicative of a spinal cord lesion, (1) represents unaffected gait with pain at paravertebral palpation, (2) refers to ambulatory paraparesis, (3) non-ambulatory paraparesis, (4) paraplegia with intact DPP, and (5) paraplegia with absent DPP.

**Fig. 2:**
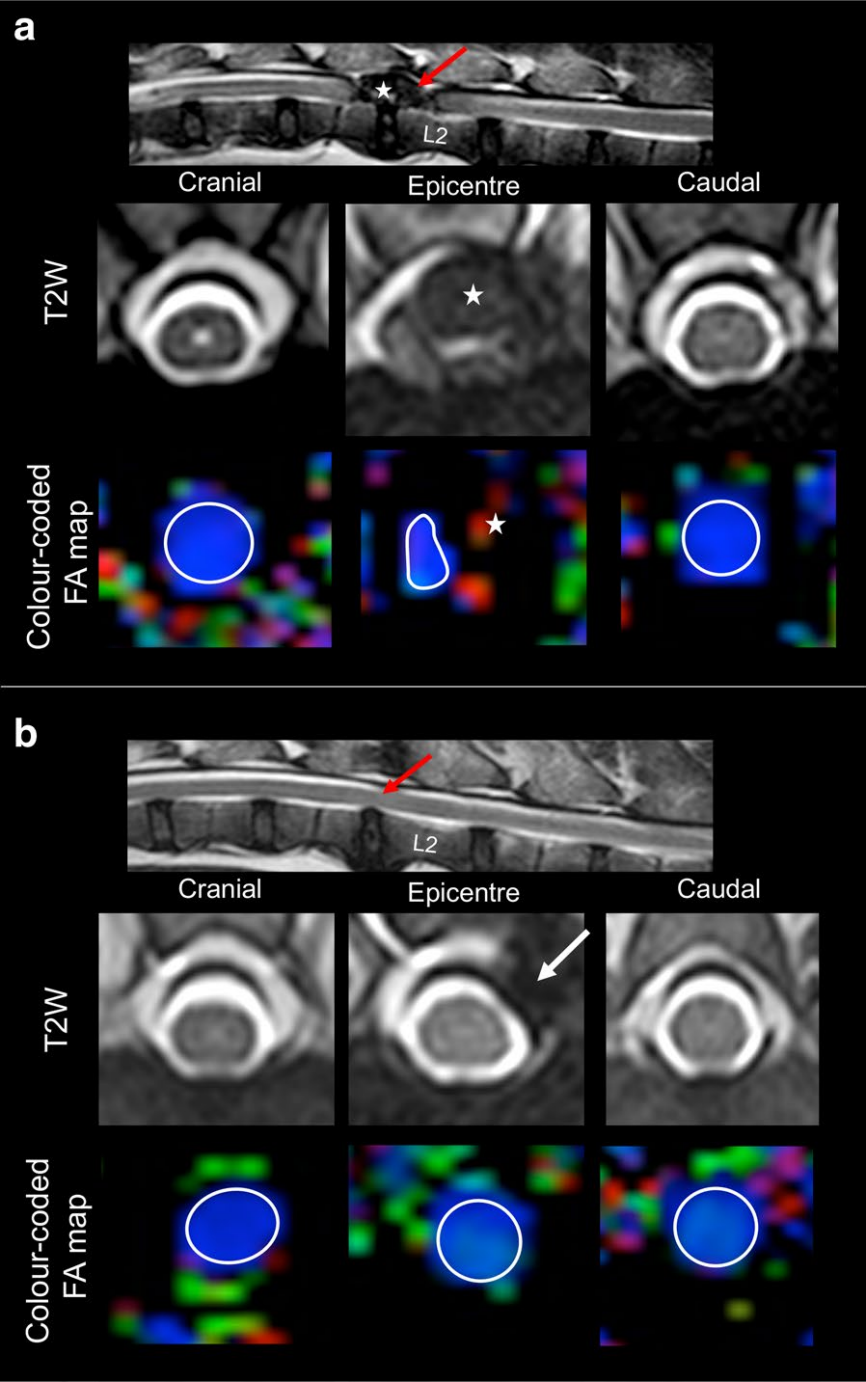
Selection of regions of interest. Sagittal and transverse T2W images and transverse colour-coded FA maps of the spinal cord of a 17.7 kg mix-breed male dog, 6.6 years-old, with acute onset of paraplegia due to an intervertebral disc herniation (IVDH) at the level of L1–2 before (**a**) and 12 weeks after decompressive surgery (**b**). The red arrow points at the epicentre of lesion in sagittal T2W images. The star in A shows the degenerated disc material compressing the SC. The white arrow in B shows the hemilaminectomy defect performed to achieve SC decompression. Colour coding of FA maps: blue depicts craniocaudal diffusion axis, green and red indicate ventrodorsal and laterolateral diffusion axis, respectively

For DTI image processing, the software Extended MR workspace^®^ (Version 2.6.3.4, 2012, Philips Medical Systems, the Netherlands) was used. T2W and T2* images were evaluated by board certified neurologists (AT and VMS) in order to determine the localization of the IVDH for subsequent surgical approach. Additionally, these T2W images served as a baseline for anatomical land marking for the DTI. As previous reports evidenced that transversal DTI sequences minimize partial volume effects in comparison to sagittal sequences [36], regions of interest (ROIs) were placed at the defined localizations directly in the transversal colour-coded FA maps (Fig. 2). Since FA represents a normalized and rotational invariant value [48], colour coding was generated based on a combination of tensor anisotropy and direction within each voxel. In order to reduce measurement errors deriving from signals of surrounding tissues such as cerebrospinal fluid or epidural fat, the application tool “Multiple ROIs” was used to set adjacent individual voxels within the white and grey matter of the SC in a transversal view. These voxels were afterwards fused to form a single ROI as previously described; all ROIs were placed on signal deriving from the SC tissue directly dorsal to intervertebral disc spaces. Lesion epicentres were defined as localizations of spinal cord compression caused by herniated disc material in T2W sequences [39]. ROIs were placed directly at the epicentre and one spinal cord segment adjacent to any compression (cranially and caudally). FA and ADC values were gathered from each ROI.

### Statistical analysis

Diffusion tensor imaging metrics of the control population were calculated using mean values of at least two ROIs placed in the SC caudally of the twelfth thoracic vertebra and cranially of the third lumbar vertebra.

Measurements of FA and ADC values were compared between preoperative and follow-up scans, as well as between dogs suffering from IVDH and controls by means of t-tests. Comparisons among the different localizations, in the lesion epicentre, cranially and caudally of the lesion, were performed using a multiple analysis of variance and a Tukey–Kramer adjustment. The assumption of normality was tested by means of a Kolmogorov–Smirnov test and visual assessment of qq-plots of model residuals. Covariance analyses were additionally performed with each variance analysis to evaluate the effect of body weight and age between groups. Furthermore, Spearman tests were conducted to assess correlations between DTI metrics measured at the epicentre of the SCI (continuous variable) and clinical scores after decompressive surgery (ordinal scaled variable). For this purpose, the commercially available software SAS^®^, version 9.2 (SAS Institute, Cary, NC, USA) and GraphPad Prism^®^ (version 5, GraphPad Software, CA, USA) were used for the statistical calculations and graphic elaboration, respectively. Significance level was considered when p < 0.05.

## Results

### Dogs

Seventeen paraplegic dogs, 7 females and 10 males, affected by acute SCI caused due to IVDH were included. The SCI dogs had a mean age of 5.5 years (median = 4.6 years; range 2.2–13.1 years) and a mean body weight of 9.9 kg (median = 8.6 kg; range 3.8–19.6 kg). Dachshunds (n = 5) and mixed-breed dogs (n = 5) were the most common. Moreover, two Jack Russell terriers, two Shih Tzu, and one individual of each of the following breeds were recruited: Havanese, small Munsterlander pointer, and French bulldog. The mean time between onset of non-ambulatory status and preoperative MRI examination was 1 day (median = 1 day; range 0–6 days). The most commonly affected intervertebral disk spaces were Th12/13 and Th13/L1. Twelve dogs showed a response to nociceptive stimulation and 5 dogs showed no presence of DPP in the pelvic limbs. Presence of intramedullary signal voidance in gradient-echo T2* sequences suggesting intramedullary haemorrhage could not be evidenced in any case. All 17 paraplegic dogs underwent surgical decompression of the spinal cord immediately after MRI and regained motor function within 4 weeks thereafter. Follow-up MRI examination was performed 12 weeks after first noticing MFR and at this time point, all dogs were able to walk, one of them with support. In one dog with clinical improvement the follow-up scan could not be performed. Clinical grading at both time points is depicted in Table 1.

**Table 1:** SCI dog characteristics

Clinical grade	At presentation (n = 17)	At follow-up examination (n = 16)
0	–	4
1	–	–
2	–	11
3	–	1
4	12	–
5	5	–

*SCI* spinal cord injury; clinical grading. 0: dog with no neurological deficits; 1: hyperaesthesia with paravertebral palpation; 2: ambulatory paraparesis; 3: non-ambulatory paraparesis; 4: paraplegia with deep pain perception; 5: paraplegia without deep pain perception

### Fractional anisotropy

**Fig. 3:**
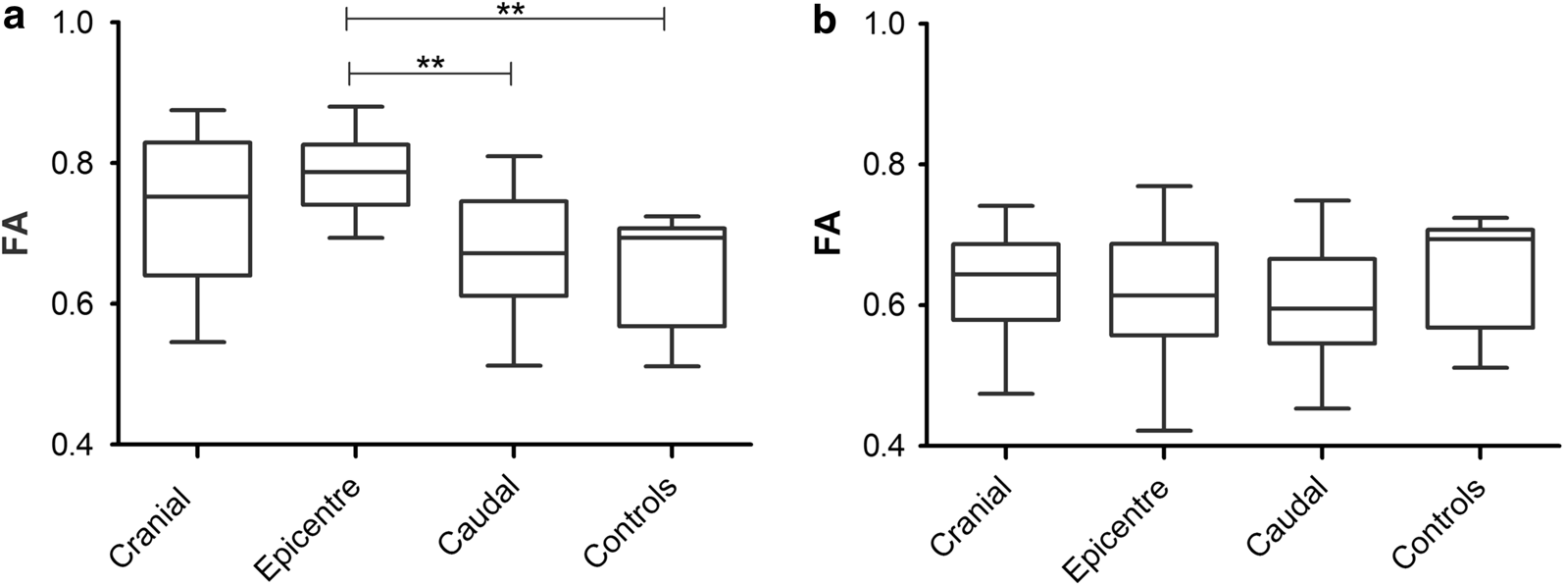
Distribution of FA values. Tukey boxplots depicting the distribution of fractional anisotropy (FA) values at each localization before (**a**) and 12 weeks after showing motor function recovery (**b**). Values at epicentres showed significant increases compared to controls and perilesional values measured caudal to the epicentre. No differences were found in the follow-up MRI examination

Before decompressive surgery, values of FA at the site of the lesion epicentre in paraplegic dogs with acute SCI were higher than in the controls (p = 0.0097). At the same time point, FA values at epicentres were significantly higher compared to the values in the SC segment one vertebral body caudally (p = 0.0014; Fig. 3a). T-tests performed between time points revealed a significant higher FA value before surgery at each localization (Table 2). Three months after MFR, FA showed no statistical difference when compared with the control group (Fig. 3b). Covariance analysis performed for the assessment of the effect of age and body weight found no differences among groups at both time points (Additional file 1: Table S1). Clinical scores 12 weeks after MFR did not correlate to FA values in the epicentre at the same time point (p = 0.39).

**Table 2:** Temporal evolvement of DTI metrics after spinal cord decompression

Diffusion metrics	SC segment	At presentation (n = 17)	Follow-up (n = 16)	*p* value
FA; median ± SD	Cranial	0.725 (± 0.105)	0.627 (± 0.075)	*0.0025*
	Epicentres	0.781 (± 0.053)	0.611 (± 0.092)	*< 0.0001*
	Caudal	0.678 (± 0.087)	0.604 (± 0.074)	*0.0049*
ADC (10^−3^mm^2^/s); median ± SD	Cranial	0.999 (± 0.317)	1.224 (± 0.575)	*0.0156*
	Epicentres	0.817 (± 0.236)	1.134 (± 0.366)	*0.0016*
	Caudal	0.985 (± 0.197)	1.198 (± 0.507)	0.0646

Italic values indicate significance of *p* value (*p* < 0.05)

Epicentres: ROIs placed in spinal cord compressed by herniated nucleus pulposus material, directly above the respective intervertebral disc space. Cranially: ROIs placed in spinal cord one vertebral body cranially to epicentres. Caudally: ROIs placed in spinal cord one vertebral body caudal to epicentres

*DTI* diffusion tensor imaging, *FA* fractional anisotropy, *ADC* apparent diffusion coefficient, *SD* standard deviation, *SC* spinal cord

### Apparent diffusion coefficient

**Fig. 4:**
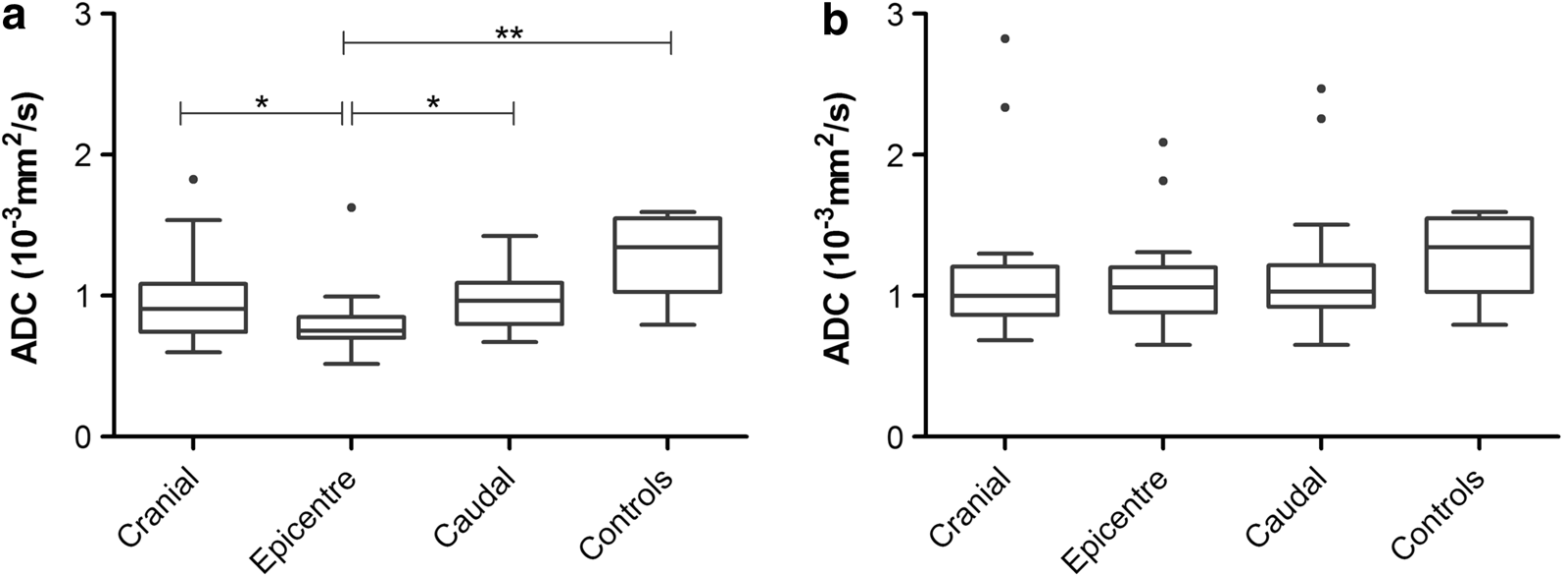
Distribution of ADC values. Tukey boxplots depicting the distribution of apparent diffusion coefficient (ADC) values at each localization before decompressive surgery (**a**) and at follow-up scan 12 weeks after MFR (**b**). Before decompressive surgery, values gathered from the epicentre were significantly lower than that of controls. Epicentres displayed lower values than values cranially and caudally. At follow-up scans, ADC values from dogs with MFR did not differ from controls

In the acute stage before decompressive surgery, ADC values were lower in the epicentres compared to ROIs set one spinal cord segment cranially and caudally (p = 0.0448 and p = 0.0433, respectively; Fig. 4a). Moreover, ADC values derived from the epicentre of the lesion were significantly lower in dogs with acute contusive-compressive SCI than in control dogs (p = 0.0035; Fig. 3a). Temporal evolvement of ADC values could be evidenced with t-tests performed between pre-operative measurements and follow-up scans. ADC values were significantly lower in the compressed spinal cord than in the follow-up status at the epicentre and cranially (p = 0.0016 for epicentres; p = 0.0156 cranially; Table 2). Additionally, no significant differences in ADC values could be found between SCI affected dogs and controls at follow-up examinations (Fig. 4b); neither age nor body weight showed an effect in variance analysis performed among groups for the pre- and postoperative time points (Additional file 1: Table S1). Clinical scores correlated negatively with diffusivity present at the epicentre of SCI in follow-up examinations (p = 0.0023; r = − 0.705).

## Discussion

The clinical approach to acute traumatic SCI faces substantial challenges including the fact that current techniques to assess severity and recovery rate are non-quantitative [49]. In this prospectively designed study, temporal evolvement of DTI values is described from the SC of paraplegic dogs with acute SCI showing MFR subsequent to decompressive surgery. The population of dogs presented and treated for acute SCI caused by IVDH match with previous reports, being mostly middle-aged dogs of chondrodystrophic breeds [50–52]. Furthermore, localization of disc herniation within the vertebral column occurred at the most commonly reported sites [22, 53, 54].

Limited information is available regarding DTI in the course of acute SCI in humans. Albeit elevations of FA values have been reported after acute onset of clinical signs [55], a reduction of FA values in the epicentre of the lesion seems to occur more commonly [56, 57]. In humans, dural laceration occurs concomitantly with contusion and/or compression of the SC, as consequence of vertebral fractures or luxations. Such laceration is more commonly observed in humans than in dogs and could be responsible for primary transection of axonal membranes with associated intramedullary influx of CSF and haemorrhage fluids, subsequently leading to alterations in the intra- to extracellular water content and therewith decreasing anisotropy [9, 55–57]. Conversely, an increase in anisotropy at the epicentre of the lesions occurred, which suggests presence of cytotoxic oedema and reduced space between axonal tracts caused by a reduction of the diameter of the vertebral canal due to the presence of herniated disc material during the acute phase [39, 58, 59]. Furthermore, increases of FA values have been proposed to be more dependent on changes of cellular membranes than on myelin sheaths and have been therefore proposed as a biomarker for cytotoxic oedema during the acute phase of axonal injury after traumatic brain lesions [28, 59–61].

Diffusivity changes depicted by decreased ADC were found in epicentres of the SC in dogs before undergoing decompressive surgery compared to control values. Previous studies describing histopathologic changes in canine acute SCI showed that intra-axonal ultrastructural changes such as disarrangement of axoplasmic neurofilaments and mitochondrial accumulation occur predominantly at the lesion epicentre, although distant segments away from the compression site were also but less severely affected [14, 21]. Therefore, low diffusion magnitude found in dogs with SCI seems to be an indicator of such intracellular damage. Additionally, significantly lower ADC values at the epicentre compared to ADC values of the SC one vertebral body cranially and caudally suggest a complementary distorted diffusivity caused by mechanical compression and permanent deformation exerted by the extruded disc material on the spinal cord at the time of the preoperative MRI scan.

At follow-up examination 12 weeks after evidence of MFR, both, FA and ADC values showed no differences when compared to those of SC of control individuals, indicating absence of contusive-compressive forces or massive reduction of intraparenchymal architecture that could cause abnormal restriction of magnitude or direction of water molecule diffusion because of accordance between diffusion metric tendency to normality and MFR of the dogs.

Since the majority of dogs acutely affected with SCI had an intact DPP at presentation at the clinic, a relative conservation of tissue architecture after moderate to severe SCI may explain that FA and ADC values tend to normality after prompt surgical intervention. Moreover, timely decompression of the spinal cord could have led to effective reperfusion of parenchyma, thereby avoiding possible worsening of clinical signs by preventing further tissue damage. Additionally, the fact that no differences in DTI metrics were found between SCI affected dogs with recovered motor function and controls may support that complex intrinsic reparatory mechanisms take place within the canine SC days after SCI caused by IVDH. Examples of such mechanisms are expression of Growth Associated Factor-43 (GAP-43) as indicator of axonal regeneration and remyelination accomplished by Schwann cells and oligodendrocytes playing an important role in microarchitecture preservation and remodelling [14, 21].

Interestingly, lower ADC values correlated with higher clinical grades in the follow-up examination. A possible interpretation of this result could be that, although directionality of diffusion was restored after decompression, intra-axonal ultrastructural changes inherent of the secondary injury such as mitochondrial accumulation may still represent a long-lasting effect on diffusion magnitude [14, 21]. Nevertheless, information regarding histopathological characterization of the spinal cord during motor function recovery is limited and this correlation derives from a relatively small population of dogs and therefore should be carefully interpreted.

As all dogs recruited in this study were still alive at the time of its completion, the lack of histopathological and immunohistochemical studies of epicentres and perilesional SC segments represents a limitation. However, Yoon and colleagues recently found a correlation between histopathologic findings and DTI metrics in dogs with experimentally induced SCI [38]. Moreover, normal anatomical structures of the spinal cord are displaced and deformed by the extruded intervertebral disc material during IVDH; therefore, a clear distinction between white and grey matter, as well as visualization or evaluation of diffusion metrics of individual funiculi using clinical applicable protocols in the canine spinal cord is still beyond the study’s scope.

Establishing quantitative methods that objectively evaluate the recovery phase after SCI is mandatory for treatment studies [5]. Performing DTI during MFR in people affected by acute compressive SCI is challenging, as the vertebral column fractures are often stabilized with metallic implants being a source for artefacts in MRI scans [62–64]. For this reason, DTI in naturally-occurring canine SCI represents a unique opportunity to understand microstructural changes of the spinal cord during MFR in a large animal translational model.

## Conclusions

In conclusion, abnormal FA and ADC values evident at the epicentre of the acutely compressed spinal cord in paraplegic dogs and reflecting distortion in water molecule diffusion are normalised 12 weeks after MFR. The present study represents therefore a basic instrument for studies evaluating effects of novel therapeutic interventions, since objective data might be gathered on a microstructural level in vivo using this technique.

## Additional file

**Additional file 1: Table S1.** Covariance analysis evaluating the effect of age and body weight in variance analysis between affected dogs and controls.

## Abbreviations

ADC: apparent diffusion coefficient
CSF: cerebrospinal fluid
DPP: deep pain perception
DTI: diffusion tensor imaging
DWI: diffusion weighted imaging
FA: fractional anisotropy
FOV: field of view
IVDH: intervertebral disc herniation
MFR: motor function recovery
MRI: magnetic resonance imaging
ROI: region of interest
SCI: spinal cord injury
SE: spin echo
TE: echo time
TR: repetition time
