# Supplementary material for: The role of diffusion tensor imaging as an objective tool for the assessment of motor function recovery after paraplegia in a naturally-occurring large animal model of spinal cord injury
Source: J Transl Med. 2018 Sep 17;16:258. doi: 10.1186/s12967-018-1630-4 (PMC6142343; doi:10.1186/s12967-018-1630-4)
Supplement: Supplementary file 1 — Additional file 1: Table S1. Covariance analysis evaluating the effect of age and body weight in variance analysis between affected dogs and controls. [file 12967_2018_1630_MOESM1_ESM.docx]

**Table S1.** Covariance analysis evaluating the effect of age and body weight in variance analysis between affected dogs and controls.

|  | FA | ADC |
| --- | --- | --- |
| Covariances first time point of evaluation |  |  |
| Age | 0.0635 | 0.3489 |
| Body weight | 0.4458 | 0.0928 |
| Covariances follow up examination |  |  |
| Age | 0.8138 | 0.1625 |
| Body weight | 0.7055 | 0.1256 |

FA = Fractional anisotropy; ADC = apparent diffusion coefficient
